# Killing Hypoxic Cell Populations in a 3D Tumor Model with EtNBS-PDT

**DOI:** 10.1371/journal.pone.0023434

**Published:** 2011-08-18

**Authors:** Conor L. Evans, Adnan O. Abu-Yousif, Yong Jin Park, Oliver J. Klein, Jonathan P. Celli, Imran Rizvi, Xiang Zheng, Tayyaba Hasan

**Affiliations:** 1 Wellman Center for Photomedicine, Harvard Medical School, Massachusetts General Hospital, Boston, Massachusetts, United States of America; 2 Department of Chemical and Biomolecular Engineering, Korea Advanced Institute of Science and Technology, Daejeon, Korea; National Cancer Institute, United States of America

## Abstract

An outstanding problem in cancer therapy is the battle against treatment-resistant disease. This is especially true for ovarian cancer, where the majority of patients eventually succumb to treatment-resistant metastatic carcinomatosis. Limited perfusion and diffusion, acidosis, and hypoxia play major roles in the development of resistance to the majority of front-line therapeutic regimens. To overcome these limitations and eliminate otherwise spared cancer cells, we utilized the cationic photosensitizer EtNBS to treat hypoxic regions deep inside *in vitro* 3D models of metastatic ovarian cancer. Unlike standard regimens that fail to penetrate beyond ∼150 µm, EtNBS was found to not only penetrate throughout the entirety of large (>200 µm) avascular nodules, but also concentrate into the nodules' acidic and hypoxic cores. Photodynamic therapy with EtNBS was observed to be highly effective against these hypoxic regions even at low therapeutic doses, and was capable of destroying both normoxic and hypoxic regions at higher treatment levels. Imaging studies utilizing multiphoton and confocal microscopies, as well as time-lapse optical coherence tomography (TL-OCT), revealed an inside-out pattern of cell death, with apoptosis being the primary mechanism of cell killing. Critically, EtNBS-based photodynamic therapy was found to be effective against the model tumor nodules even under severe hypoxia. The inherent ability of EtNBS photodynamic therapy to impart cytotoxicity across a wide range of tumoral oxygenation levels indicates its potential to eliminate treatment-resistant cell populations.

## Introduction

The last several decades have seen countless advances in our ability to detect and treat cancer. However, the mortality associated with certain cancers, such as ovarian, remains unacceptably high. Ovarian cancer (OvCa) represents a major therapeutic challenge, as tumors too often evade front-line treatments, causing the majority of patients to eventually succumb to treatment-resistant disease. Problematically, OvCa is typically diagnosed at an advanced stage, where patients present with disseminated metastatic lesions coating the surfaces of the peritoneal cavity. It is impossible to remove all lesions surgically, leaving behind significant sub-millimeter sized residual tumors. Women with recurrent, resistant OvCa face a poor quality of life and a dismal five-year survival rate of 30% [1].

In an attempt to eradicate these tumors, physicians turn to a toolkit of therapies that can be tailored for specific cancers, and increasingly, specific patients. Combination OvCa regimens, such as carboplatin-paclitaxel therapy, have resulted in moderate patient survival improvements [1], with intraperitoneal (*i.p.*) administration currently being the preferred method at most institutions. However, numerous factors can limit the delivery of *i.p.* therapeutics into OvCa nodules, including the structure and charge of the agent, physiological factors such as permeability, and the presence of tumor stroma, rich in extracellular matrix (ECM) [2]. Intravenous therapeutics also suffer from limited uptake as tumors have disorganized, leaky, and branched vessel networks with high interstitial pressure [3], [4].

Poor tissue perfusion and diffusion leads to the hypoxia observed in tumors [5], [6] such as metastatic OvCa lesions [7]. Diffusion typically allows for only ∼70 µm of gas penetration, resulting in widespread oxygen-depleted tumor regions [8]. Hypoxia triggers an array of cellular defense mechanisms, many of which are activated by the hypoxia-inducible transcription factor 1, that protect hypoxic tumor cells from potent therapies [9]. These mechanisms render front-line agents for OvCa, such as carboplatin and paclitaxel, ineffective in hypoxic environments [10], with paclitaxel demonstrated to be over 100 times less effective under hypoxic conditions [11]. Without the ability to produce radicals through oxygen consumption, radiation therapy also loses its cytotoxic potential under hypoxia [9]. Furthermore, anaerobic glycolysis causes tumor acidification [12], [13], substantially altering the uptake of chemotherapeutics and conferring chemoresistance against certain agents [14]. Therapeutics, therefore, often inadequately treat subpopulations of cancer cells, leading to the proliferation of chemorefractive or chemoresistant disease. To eliminate this surviving population, it is imperative to expand the physicians' treatment toolkit with regimens designed to target normally protected tumor cells. Such therapeutic agents must pass through the ECM, rapidly permeate through cell layers, and uptake into cells. Importantly, any therapeutic needs to be highly effective in both normoxic and hypoxic environments, and must evade cancer cells' typically up-regulated anti-apoptotic machinery.

Treatments based on photodynamic therapy (PDT) have the ability to address these requirements. An FDA-approved cancer treatment, PDT uses molecules known as photosensitizers that generate cytotoxic reactive species when exposed to particular wavelengths of light [15]. PDT is particularly apt for cancer treatment as photosensitizers selectively accumulate in cancerous lesions and the therapy is limited only to irradiated tissues. PDT of OvCa patients using the first generation, non-specific photosensitizer Photofrin, which although suboptimal, was found to confer a significant survival advantage [16]. The second-generation photosensitizer BPD (benzoporphyrin derivative monoacid A) showed promise *in vivo* [17] and has been found to be synergistic with chemotherapy [18] and biological therapies [19]. PDT with BPD has been observed to be effective even against drug-resistant OvCa [20]. A major drawback using these porphyrin-based photosensitizers, however, is that they also suffer from penetration problems and require molecular oxygen to impart cytotoxicity, meaning that hypoxic tumor regions are still protected against these powerful agents.

Building upon early studies with cationic phenothiazinium molecules, this work demonstrates that PDT is capable of effectively treating deep, hypoxic tumor cell populations when using a molecule from a different class of photosensitizers. Here we use the small cationic, methylene blue-like molecule EtNBS (5-ethylamino-9-diethyl-aminobenzo[a]phenothiazinium chloride), which is rapidly taken up by cancer cells and malignant tissues and achieves maximum concentration in tumors at 3 hours following subcutaneous injection [21], [22]. While EtNBS concentrates primarily in lysosomes, upon activation with red light (640–660 nm) it can burst out of these compartments and react throughout the cell [23]. EtNBS-PDT has been studied and found safe in animal experiments where it was observed to confer significant treatment benefits [22], [24]. EtNBS can mediate its phototoxicity through a molecular-oxygen dependent, singlet oxygen mediated (“Type II”) mechanism with 3% efficiency [25].

Importantly, EtNBS is also able to impart cytotoxicity without consuming oxygen (“Type I” photosensitization), making it an attractive therapy for treating cellular subpopulations of hypoxic cancer cells. In this mechanism, the excited molecule creates cytotoxic radical species by directly interacting with biomolecules and water, creating lipid and protein radical species, as well as hydroxyl radicals and superoxide species [22]. These cytotoxic moieties diffuse and react with surrounding organelles and cellular structures, triggering apoptosis. This gives EtNBS the ability to mediate its cytotoxic effects through two different photochemical channels, which is of critical importance when treating cells in both normoxic and hypoxic environments. This property of EtNBS has not yet been exploited to treat therapeutically-resistant tumor regions. By combining the treatment of 3D *in vitro* models of OvCa with optical imaging approaches, here we show that EtNBS can effectively treat otherwise protected and unresponsive cellular populations.

## Results

### The 3D Model Recapitulates Physiological Treatment Resistance Mechanisms

**Figure 1:**
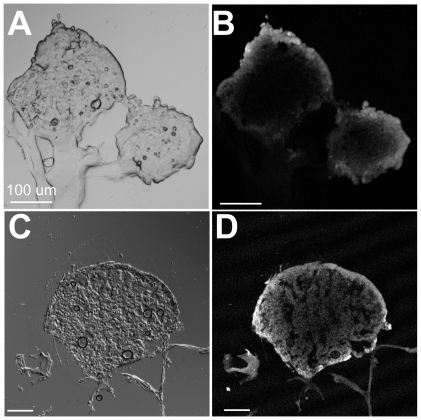
Uptake of the photosensitizer BPD over 1.5 and 4.5 hours. Images (A) and (B) are from a sample incubated with BPD for 1.5 hours; images (C) and (D) are from a 4.5 hour incubation. (A) Transmission image of two OvCa nodules following cryosection. The thickness of the slice was 30 µm. Matrigel can be seen attached to the nodules in the bottom-left portion of the image. (B) Confocal image of BPD fluorescence in the nodule, revealing its limited uptake into the nodules. (C) DIC image of a similarly prepared 30 µm slice through an OvCa nodule. (D) BPD fluorescence image, showing that even after 4.5 hours, the majority of BPD is retained in the periphery of large nodules.

Monolayer culture systems are useful in providing insight into cellular-level treatment response, with animal models often used to validate therapeutic efficacy on a large scale. Both approaches, however, can miss critical time-sensitive and spatial information. In contrast, 3D *in vitro* tumor models provide many critical cell signaling cues to recapitulate *in vivo* features while retaining the capability for real-time, cellular-level imaging for therapeutic uptake and response. The 3D adherent OvCa model used in this study was specifically created to model the multitude of avascular metastatic tumors that coat surfaces of the peritoneal cavity [18], [26], [27]. In this system, OvCa cells were cultured on the surface of an ECM-rich gel bed (Matrigel), and developed into nodules varying in sizes over the course of days. Previous studies using such *in vitro 3D* models have predominately utilized small adherent 3D cultures (40–150 µm diameter), which do not adequately address clinically-relevant treatment deficits such as limited drug penetration and hypoxia [1]. To probe these critical problems, we grew the adherent OvCa model for a period of 13 days to achieve a population of large nodules that are >200 µm in diameter [26]. Poor diffusion into large nodules is a major impediment in Platin-based therapy, where compounds such as cisplatin were found to localize at the nodule periphery, with much lower concentrations in the nodule core [28]. In our 3D OvCa model, we found that the anionic photosensitizer BPD diffused weakly into nodules during 1.5 hours of incubation, primarily concentrating in the nodule periphery with a penetration depth about 125 µm (Figures 1A,B). Even after an incubation period three times this standard duration, the majority of BPD was still localized on the nodule surface (Figures 1C,D), sparing cells deep in the model tumor.

**Figure 2:**
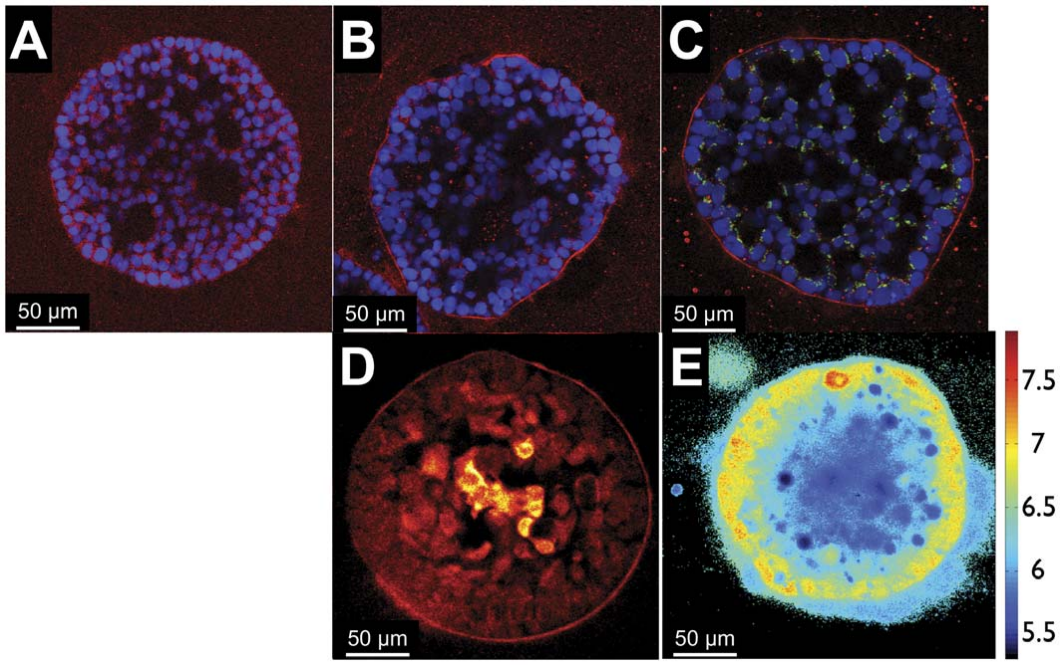
Physiological factors can cause poor therapeutic outcomes in metastatic OvCa nodules. Large nodules stained for the presence of ECM show coatings of human fibronectin (A), collagen IV (B), and laminin V (C), in red. Cell nuclei were stained with DAPI (blue). Staining with the Golgi protein GM130 (C, green) reveals that the outer nodule cell layer is polarized. Multiphoton microscopy of PIM-stained, day 13 nodules reveals the presence of hypoxic core cells (D, false color). pH imaging using SNARF-4F shows that the core of the model nodules are acidic (E). Autofluorescence gave rise to the two deeply acidic (pH<5.5) point artifacts in the center of the image.

One component that limits therapeutic penetration is the extracellular matrices (ECM) that are present in the tumor microenvironment. The 13-day-old OvCa nodules were fixed and stained for three common ECM proteins that are important in metastatic OvCa: fibronectin, collagen IV, and laminin V [29]. As seen in Figures 2A–C, all three ECM proteins are observed to coat the exterior of the nodule, forming a physical barrier to therapeutic delivery. GM130 staining of the Golgi apparatus reveals that the outermost layer of cells in contact with the ECM share a common apical polarity, while cells in the center of the nodule show random polarity. This apical polarity is an organizational feature observed in 3D *in vitro* tumor cultures and is thought to be due to the interaction of cells with the coat of the ECM proteins [30]. This ECM barrier most likely plays a critical role in the penetration of platin chemotherapeutics, and traps BPD at the nodule surface, as seen in Figure 1D.

Even if therapeutics can penetrate into the nodule, their efficacy can still be severely hampered by the presence of hypoxia. Oxygen diffusion studies indicated the possibility that the OvCa nodules likely had hypoxic cores [31]. By imaging nodules stained with pimonidazole using two-photon microscopy, we found a consistent pattern of hypoxia throughout the cores of our *in vitro* OvCa nodules larger than 200 µm in diameter (Figure 2D). The pH gradients caused by hypoxia-induced acidification of the tumor microenvironment can also have a major effect on treatment by altering the partitioning and uptake of therapeutics into cells [32]. pH gradients in living OvCa nodules were visualized using the spectral-ratiometric pH imaging agent SNARF-4F (Figure 2E). A clear pH gradient was observed along the radii of nodules, with the pH ranging from neutral at the nodule periphery to levels below six in nodule cores. These acidic cores were consistently observed across both small and large nodules, indicating the widespread presence of acidosis.

**Figure 3:**
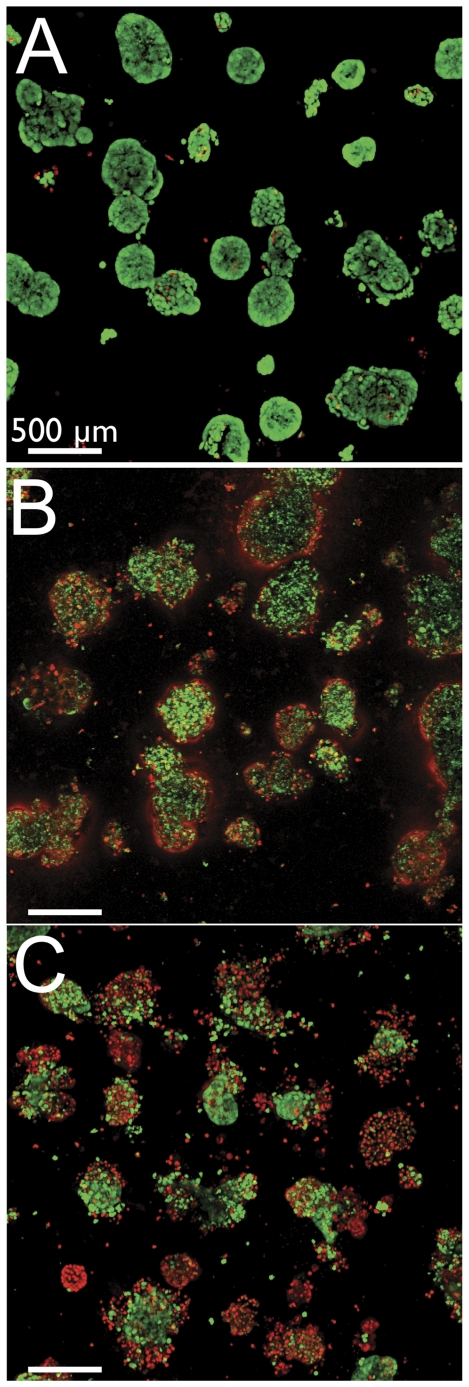
The cores of large OvCa nodules survive carboplatin treatment and BPD-PDT. (A) Live/Dead images of nodules without treatment, (B) after 72 hours of carboplatin treatment at 1 µM, and (C) following BPD-PDT at 240 nM at 10 J/cm^2^. Treated cultures both show a consistent pattern of nodular core survival. Viable cells are green, while dead cells stain red.

To visualize the impact of these therapeutic limitations in the 3D OvCa model, the large nodules were treated with either carboplatin or BPD-PDT. The nodules were observed to have spatially conserved patterns of cytotoxicity (Figure 3). Nodules treated with carboplatin were observed to have only peripheral cellular death leaving a surviving core (Figure 3B), consistent with earlier studies [18], [26], [27]. PDT with the photosensitizer BPD was also observed to primarily kill cells at the exterior of large nodules, sparing nodule core cells (Figure 3C). This outside-only treatment response pattern was observed across numerous therapeutic doses (data not shown). As therapeutically-resistant cancer is understood to evolve from cells that escape treatment, this model's surviving nodular core cells likely recapitulate a subpopulation of cells *in vivo* that are protected from treatment and may develop into resistant carcinoma.

### EtNBS rapidly concentrates into nodule core cell populations

To effectively eliminate these spared nodule cell populations, we synthesized and administered the small cationic photosensitizer EtNBS to the 3D model system. Pre-treatment (dark) toxicity experiments were carried out to determine the optimal administration concentration of EtNBS. From these studies, the ideal balance between dark toxicity and effective therapeutic concentration was found at a concentration of 500 nM (data not shown).

**Figure 4:**
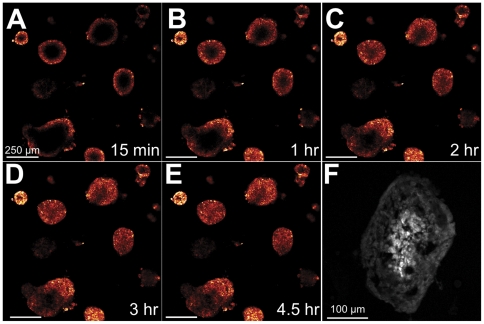
EtNBS concentrates into the cores of 3D OvCa nodules. (A–E) Time-lapse confocal microscopy images of day 13 nodules incubated with EtNBS at 500 nM. EtNBS is observed to penetrate into nodules rapidly under three hours. (F) Confocal image of a frozen, cyrosectioned nodule slice showing the actual concentration of EtNBS into the nodule core. The presence of EtNBS was confirmed using hyperspectral microscopy.

In stark contrast to the previously described treatment regimens, EtNBS was observed to rapidly diffuse throughout model metastatic nodules (Figure 4A–E, Video S1). Rapid uptake into the nodules' periphery was observed almost instantly, with EtNBS filling nodules completely within a five hour period. Importantly, the observed uptake timescale in the model nodules optimally matches what has been found safe in animal experiments, where maximum tumor concentrations were reached in three hours with a clearance time of 24 hours [22]. Based on these results, a 4.5 hour EtNBS incubation time was selected for all future experiments.

To visualize the actual (not optical) uptake pattern of EtNBS in the metastatic OvCa model, 3D cultures incubated with EtNBS were cyrosectioned into 30 µm slices. A representative image of a large nodule incubated with EtNBS for 4.5 hours can be seen in Figure 4F. Importantly, EtNBS is seen to selectively concentrate into the nodule core, the very region that typically evades therapy due to poor therapeutic uptake. This uptake intensity pattern matches nodular regions observed to be acidic (Figure 2E), suggesting that the cationic photosensitizer diffuses along the acidic pH gradient, concentrating in cells within the acidic nodule core.

### EtNBS therapy kills nodule core cells and becomes increasingly more effective with increasing nodule size

**Figure 5:**
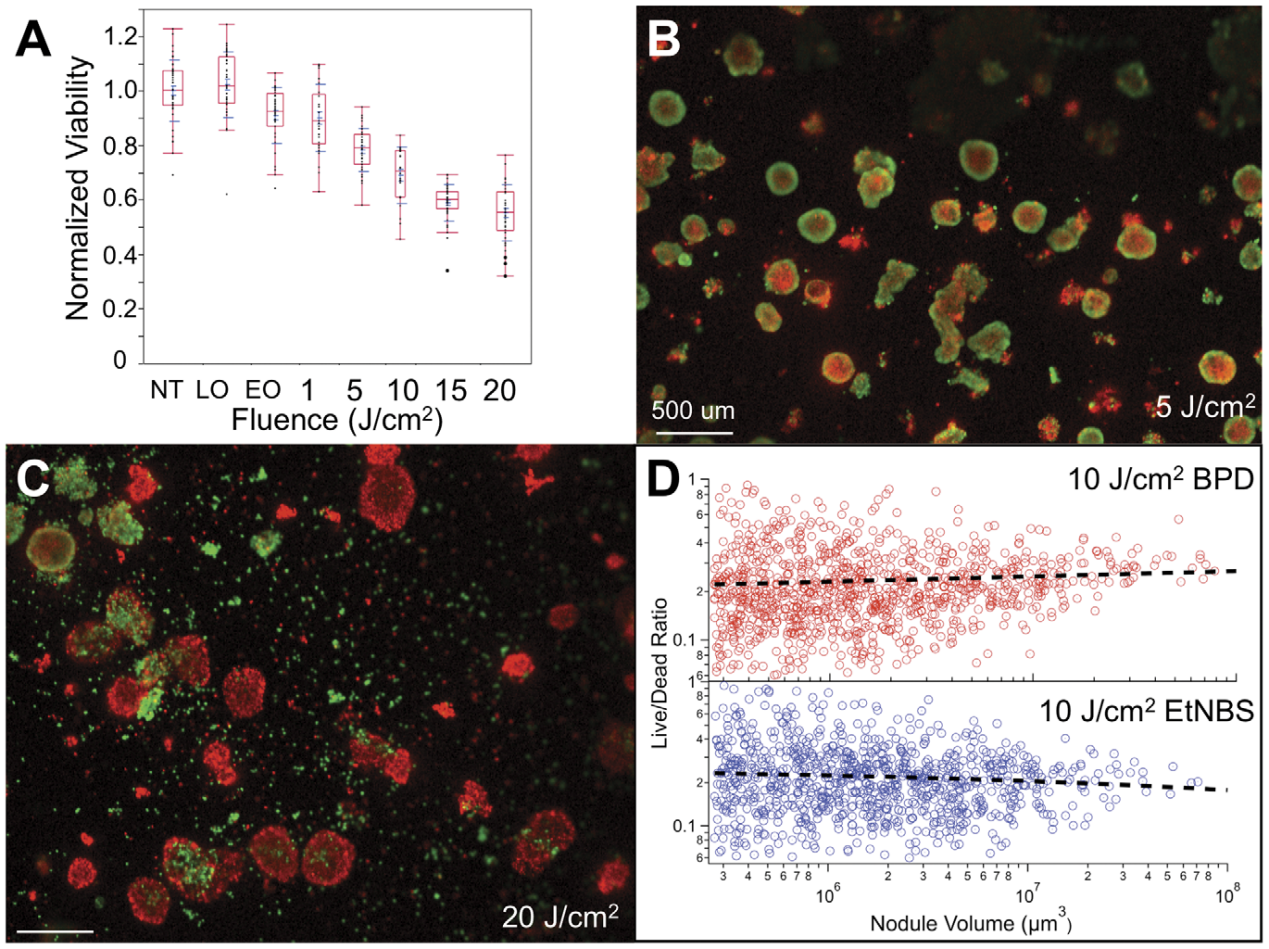
Treatment response of OvCa nodules to EtNBS-PDT. (A) Bar plot of day 13 nodule viability following EtNBS-PDT across a range of light doses. (B) Live/Dead imaging of day 13 nodules treated with 5 J/cm^2^ of 652 nm light, showing nodule core cytotoxicity. (C) Day 13 nodules treated at 20 J/cm^2^. (D) Scatter plots of nodule live∶dead ratio vs. volume of thousands of individual BPD and EtNBS PDT treated nodules. The axes are plotted on a logarithmic scale. The black dotted lines are power-law fits to the data points provided as an aid to the eye. NT = no treatment control; LO = light only control; EO = EtNBS only, without light control.

We next investigated the efficacy of EtNBS-PDT on large model OvCa nodules. Using the live-dead viability assay, the LD50 for 13-day old nodules was found to be achieved at a light dose of 20 J/cm^2^, a fraction of what is typically used in animal studies [24] (Figure 5A). Importantly, even at the low light dose of 5 J/cm^2^, EtNBS was able to selectively destroy normally protected nodule core cells (Figure 5B). This spatial pattern of EtNBS treatment response correlates with its uptake pattern (Figure 3F), demonstrating that EtNBS is capable of both concentrating into and killing otherwise untreated, hypoxic cellular populations. Moreover, the EtNBS treatment response pattern is exactly opposite of what has been observed using the peripheral-localizing therapies carboplatin and BPD (Figure 3). At higher light doses, EtNBS-PDT is observed to trigger cellular killing across entire avascular model nodules as large as 600 µm (Figure 5C). Staining with ApoTRACE revealed that these cells treated with EtNBS-PDT died via apoptosis (Figure S1).

To explore whether the cytotoxicity of EtNBS is dependent on nodule size, cultures were grown for 7, 10, and 13 days to generate nodules across a large range of diameters from 50 to 600 µm in size. Half of the nodules were incubated with EtNBS, while for comparison, the other half were incubated with the periphery-localizing photosensitzer BPD. Nodules were then treated with PDT at a light dose of 10 J/cm^2^, and the resulting live/dead images were segmented to calculate the live∶dead ratio for each individual nodule. The efficacy of BPD-PDT is observed to decrease with nodule size, consistent with its limited penetration depth. In contrast, EtNBS's uptake and cytotoxicity in the otherwise treatment-resistant core becomes increasingly beneficial with increasing nodule size (Figure 5D), likely because larger nodules have a greater portion of their cells buried in increasingly large acidic cores.

**Figure 6:**
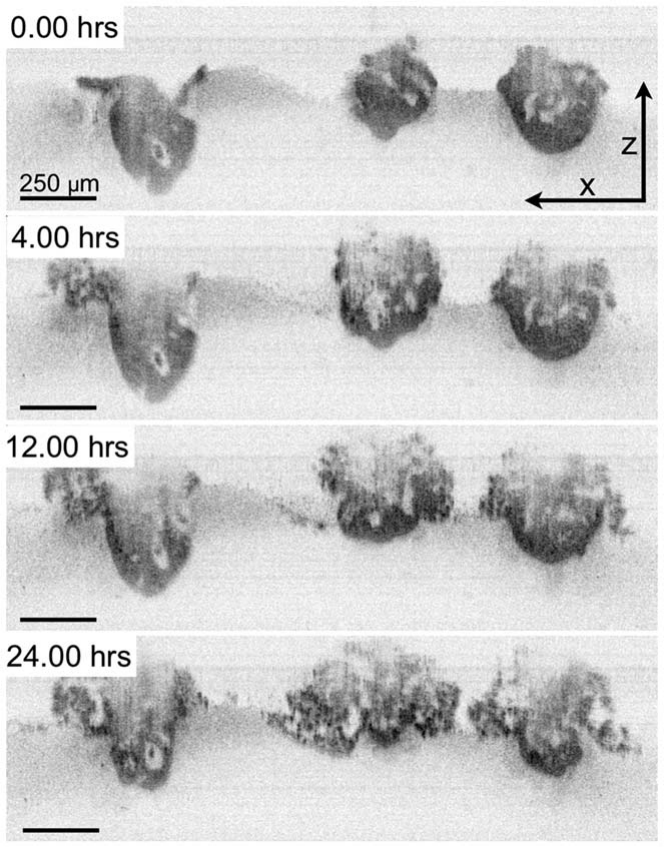
TL-OCT visualizes the treatment dynamics of large OvCa model nodules following EtNBS-PDT. Each image is one cross-sectional XZ image from the time-lapse movie (Video S2). The nodules are observed to degrade following PDT via the appearance of numerous highly scattering apoptotic bodies.

To better understand the complex treatment pattern and continuously visualize nodules' response to therapy, time lapse optical coherence tomography [26] (TL-OCT) imaging was carried out during the 24 hours immediately following EtNBS-PDT at 10 J/cm^2^. A highly sensitive cross-sectional imaging technology, TL-OCT enables label-free, non-destructive structural imaging of the large *in vitro* nodules over the course of many days. In the cross-sectional TL-OCT video, nodules are observed to immediately rupture from the inside-out within 5 hours of treatment, with numerous highly scattering apoptotic bodies pouring out of the nodules into the overlaying media (Figure 6, Video S2). This is in sharp contrast to large nodules treated with periphery-concentrating platin chemotherapeutics [26] or BPD [33], where only the nodule periphery was observed to undergo apoptosis, leaving behind surviving cores.

### The two phototoxicity mechanisms of EtNBS give rise to irradiance-dependent spatial killing patterns

**Figure 7:**
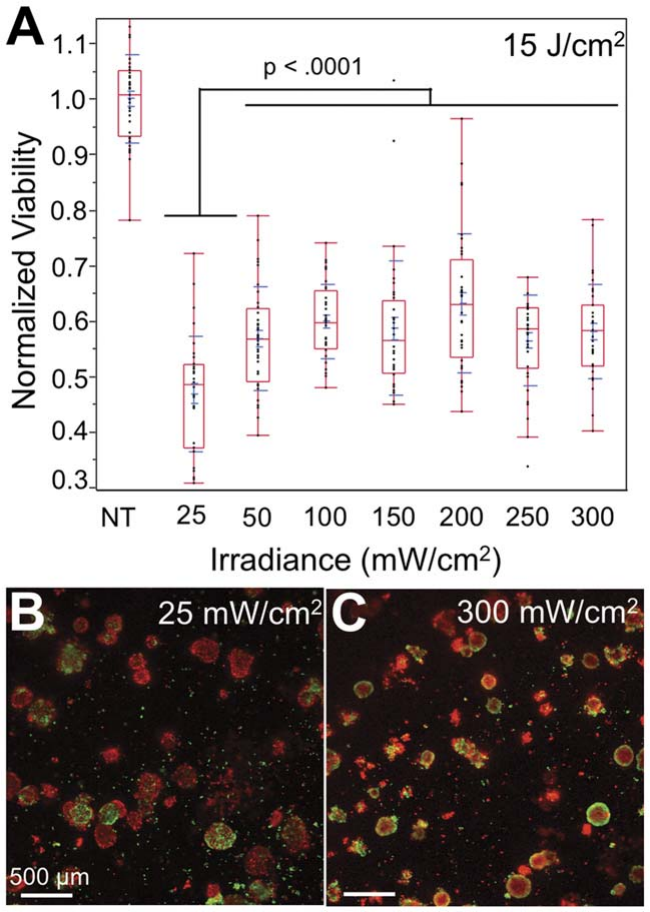
Irradiance dependence of EtNBS-PDT on therapeutic outcome. (A) Bar plot of nodule viability following 15 J/cm^2^ PDT across a range of irradiances. (B) Live/Dead image of a culture treated at 25 mW/cm^2^ irradiance. (C) Live/Dead image of a culture treated at 300 mW/cm^2^. NT = no treatment control.

EtNBS can impart cytotoxicity via two different photocytotoxicity mechanisms: the Type I, radical-mediated pathway, and the Type II, oxygen-consuming mechanism. As the Type II channel can consume and thus deplete molecular oxygen, EtNBS has the ability to treat normoxic and hypoxic cellular populations as a function of irradiance. Day 13 cultures incubated with EtNBS were treated across a range of irradiances for total fluence of 15 J/cm^2^ (Figure 7). Maximum cellular killing was observed at the lowest irradiance of 25 mW/cm^2^ and cellular viability was observed to increase with irradiance, reaching an asymptote at 100 mW/cm^2^ (Figure 7A). This decrease in treatment efficacy strongly correlates with changes in the pattern of nodule cellular death. At the low irradiances of 25 mW/cm^2^, EtNBS-PDT is capable of treating entire nodules, triggering apoptosis in both nodule cores and peripheries (Figure 7B). However, at moderate to high levels of irradiance (50–300 mW/cm^2^), when oxygen is more rapidly depleted, EtNBS is observed to instead cause only nodule core death (Figure 7C) through its primarily oxygen-independent radical-mediated pathway. This is completely different from all PDT regimens currently in clinical use, which lose their cytotoxic abilities once oxygen is consumed.

### EtNBS can impart cytotoxicity even in severely hypoxic environments

**Figure 8:**
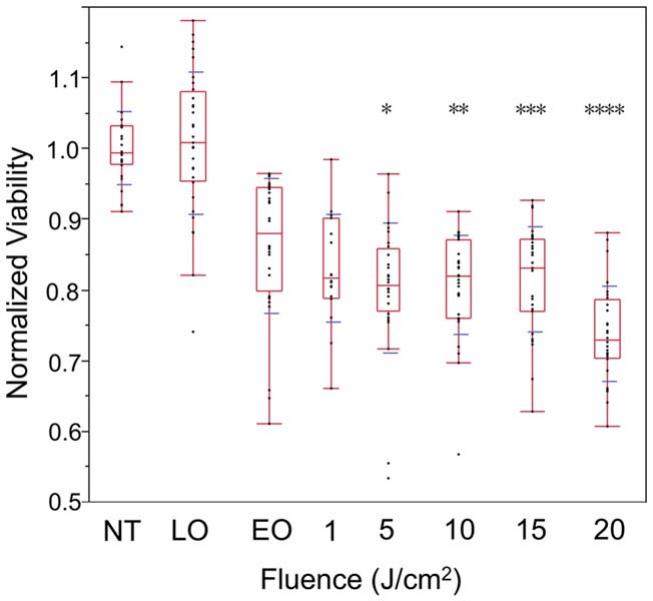
Bar plot of EtNBS-PDT treatment response from cultures incubated and treated under 100% N_2_ atmosphere. NT = no treatment control; LO = light only control; EO = EtNBS only, without light control. Asterisks indicate the statistical significance of each treatment condition when compared to the EtNBS only, no light control using a Student's t-test: * p<.005, ** p<.007, *** p<.02, and **** p<.0001.

The capability to impart cytotoxicity after oxygen depletion suggested that EtNBS may be effective in severely hypoxic or even totally anoxic environments. To test this hypothesis, day 13 nodules were incubated and treated at 100 mW/cm^2^ under 100% N_2_ atmosphere (Figure 8). Though the overall cytotoxicity of EtNBS was reduced, EtNBS-PDT was still effective under severe hypoxia, with significant cell killing observed at 20 J/cm^2^ (p<.0001). While lower fluences (5–15 J/cm^2^) were less effective, they still imparted statistically significant treatment response. The jump in cytotoxicity from 15 to 20 J/cm^2^ is not entirely surprising; PDT is a threshold phenomenon that requires a critical cellular insult to trigger apoptosis and subsequent cellular death. To determine if hypoxia plays any effects in modulating EtNBS dark toxicity, a pairwise Student's t-test was run between the dark toxicity values collected under hypoxic and normoxic conditions. No statistically significant difference was found, indicating that EtNBS dark toxicity is independent of oxygen tension.

The decrease in overall cytotoxicity may arise from the fact that Type I photochemistry can be potentiated even by low levels of O_2_ [34], which react with photoproducts to create cytotoxic radical oxygen species. This result has also been replicated under 100% CO_2_ atmosphere (data not shown), strongly suggesting that EtNBS can impart cytotoxicity in the highly acidic, severely hypoxic conditions found deep in most tumors.

## Discussion

As therapies for OvCa have developed from many careful clinical trials, the mainstay of future OvCa management will likely rest on the three major therapeutic options used today: surgery, platin therapeutics, and taxol-based treatment regimens. At the same time, years of data suggest that combination treatment regimens will be critical for future advances in battling OvCa, so it is of great importance to strategize combinations that can address specific therapeutic limitations. In this study, we show that the ability of EtNBS to penetrate deeply, concentrate into large *in vitro* nodules, and treat otherwise protected core cells even under severe hypoxia is extremely promising, demonstrating that EtNBS-PDT could be an outstanding additional tool for cancer therapy. A treatment regimen capable of working in hypoxic or even anoxic environments is extremely exciting for the treatment of OvCa and other cancers, as low pO_2_ populations are difficult to eradicate. EtNBS-based therapy is also promising because deep, hypoxic tumor cells are likely inherently different than those at the periphery. For instance, quiescent, slow-growing cells likely exist deep in hypoxic tumor compartments, which are challenging to treat with chemotherapeutics that typically target fast-growing cells. Hypoxia is known to play a fundamental role in the development of invasive and metastatic cancer as it applies selective pressure on tumor cells. Cells that proliferate in these hostile environments often have genetic predispositions for adaptive survival mechanisms that make them difficult to kill even after they migrate from the primary tumor [35]. It is also thought that cancer stem cells naturally localize into hypoxic environments, allowing them to escape current treatment regimens and repopulate the tumor with treatment-resistant cells [36]. By localizing to and effectively treating deep, acidic, and hypoxic tumor regions, EtNBS-PDT has the capability of eliminating these potentially resistant cancer cells early in the treatment process.

OvCa model nodules treated with EtNBS-PDT were observed to have irradiance-dependent treatment efficacy and spatial cellular death patterns. The data suggests that these changes arise from complementary activation of EtNBS's Type I and II phototoxicity channels, which could prove useful in clinical applications. The transition from a core-specific pattern at high irradiances to complete nodule killing (Figure 6) at low irradiances is thought to occur due to the activation of EtNBS's Type II, oxygen-consuming mechanism. At high irradiances, any oxygen-consuming photoreactions rapidly lower pO_2_, causing acute hypoxia and effectively shutting down any further Type II mediated phototherapy. At low irradiances, on the other hand, the oxygen consumption rate is far lower. When the oxygen consumption rate drops below the oxygen diffusion rate, EtNBS can react via both Type I and II mechanisms, treating both the hypoxic core cells as well as the more oxygen-enriched nodule periphery. This dual-channel property of EtNBS will be advantageous *in vivo* for treating cells present across all tumoral oxygen partial pressures, from normoxic environments to hypoxic regions.

The ability of EtNBS to localize to and kill nodule cores suggests that a combination therapy approach using EtNBS and a platin agent could be beneficial for post-resection OvCa treatment in patients. Previous OvCa model studies from Hasan and colleagues have shown that PDT with BPD, followed by treatment with carboplatin, is highly synergistic. This synergy was proposed to arise not only as the two therapies are mechanistically distinct, but also because PDT was able to disrupt nodule cellular architecture, allowing enhanced carboplatin penetration [18]. We speculate that EtNBS-PDT would likely have a similar synergy with a platin chemotherapeutic *in vivo*, especially since the two agents localize to spatially different, but overlapping, tumor microenvironments. It is thought that combination therapy will also address the small viable cellular masses observed when treating the OvCa models with high light doses (Figure 5C). These scattered cells, which have been observed in the *in vitro* model following treatment with other chemotherapeutics and photosensitizers [27], likely represent a surviving fraction *in vivo* that can be addressed through combination approaches. It is also notable that EtNBS can be administered alongside other photosensitizers like BPD for combination PDT regimens. Such a regimen was observed to be synergistic in the therapy of a mouse tumor model [37], likely because the two agents were capable of simultaneously treating different tumor regions and cellular populations.

In conclusion, we have found that the photosensitizer EtNBS is capable of not only homing into and concentrating in hypoxic tumor compartments, but can also impart substantial cytotoxicity to previously unresponsive cellular populations. PDT using a photosensitizer capable of physically penetrating deeply into tumors and working via an oxygen-independent mechanism is promising and addresses key weaknesses in many current cancer treatment regimens. These results merit further exploration in future studies using *in vivo* models of ovarian cancer. Though no published data currently supports the safe use of EtNBS in humans, future studies will focus on proving its safety and efficacy towards translating this powerful agent into clinical use.

## Materials and Methods

### In Vitro Model of Metastatic OvCa

OVCAR-5 human OvCa cells were obtained from Thomas Hamilton (Fox Chase Cancer Institute) and previously characterized by microsatellite marker analysis. Cells were grown and maintained in complete cell media comprised of RPMI 1640 (CellGrow, Mediatech) supplemented with 10% fetal calf serum (Gibco) and 1% of 5,000 I.U./mL penicillin/streptomycin (CellGrow, Mediatech). 3D cultures were plated in either 14 mm aperture coverslip-bottom Mattek plates (P35G-0-14-C, Mattek) or glass-bottomed 24 well plates (W1350, Genetix) as described previously [18], [26], [27], [38]. Cultures were maintained in 2% GFR supplemented complete media during growth periods.

### Confocal and Multiphoton Microscopy

All confocal and multiphoton imaging experiments were carried out on an inverted Olympus FV1000 hyperspectral laser scanning confocal and multiphoton microscope. A tunable Titanium Sapphire laser (MaiTai Deep See) was used for multiphoton experiments. The inverted frame housed a programmable, automated stage (ProScan II, Prior) that could accept 35 mm and multiwell plates. A temperature-controlled microincubator (Tokai-hit) was used for all confocal time-lapse imaging experiments.

### High-Throughput, High-Content Image-Based Viability Assay

To visualize the detailed *in vitro* therapeutic response, the Live/Dead viability assay (Invitrogen) was used as a high-throughput, high-content imaging assay as previously described [18], [27]. Image analysis was carried out using the Matlab programming toolkit (Mathworks) and the open-source LOCI tools. To determine the well-by-well viability, histograms were generated for the two fluorescence channels to determine the background intensity for automatic subtraction. The intensities of the pixels in each channel were then summed for a total live or dead fluorescence intensity. The viability of each well was tabulated by dividing the total live intensity by the sum of the live and dead intensities. All viabilities were normalized to no treatment (NT) control wells. Statistical analysis was accomplished using the JMP 8 software package (SAS) using pairwise Student t-tests to calculate statistical significance. To measure individual *in vitro* OvCa nodule viabilities, the fluorescence channels from each image file were background subtracted, and then summed together into a single intensity image. This image was then thresholded using Otsu's method, creating a binary image from which nodule equivalent diameters were calculated. This binary mask was then applied to the background-subtracted fluorescence channels to calculate the viability of each nodule.

### Live and Fixed Cell Imaging

The HypoxyProbe-1 kit (Hypoxyprobe, Inc.) was used to visualize hypoxia in the model nodules. Pimonidazole was added to nodules grown in Mattek dishes at a concentration of 200 µM and incubated at 37°C for 3 hours. These plates were then stained following a protocol modified from Debnath et al. [26], [30]. Nodules were incubated at room temperature overnight with the mouse anti-PIM antibody, followed by conjugation to AlexaFluor 568 goat anti-mouse secondary antibodies. Two Photon microscopy was used to image the fluorescence signal throughout the entire nodule. AlexaFluor 568 was excited at 760 nm, and the non-descanned emission was selected using a 570 nm LP dichroic mirror (Omega Optics) and filtered using a 575–630 filter (Omega Optics). Immunofluorescence staining of human fibronectin (Sigma), collagen IV (DAKO), laminin V (Millipore), and the Golgi protein GM130 (BD Transduction Labs) were also carried out and imaged using confocal microscopy. ECM antibodies were conjugated with AlexaFluor 568 labeled secondary antibodies. GM130 was labeled with AlexaFluor 488. DAPI (Sigma) was used to visualize cell nuclei.

The pH of cells in the model OvCa nodules was measured using the spectral ratiometric dye SNARF-4F AM ester (Invitrogen). 3D cultures were incubated with 20 µM SNARF-4F AM for 1 hour in the dark prior to imaging. A confocal hyperspectral image cube was collected for each nodule from 550 to 800 nm with a spectral interval of 5 nm and a bandwidth of 10 nm. A Matlab script analyzed the hyperspectral image cubes to calculate pH images by taking the ratio of the 570 and 650 nm emission bands. The calculated ratios were converted to pH levels using the pH-dependent spectra provided by Invitrogen.

### Imaging Apoptosis

Apoptosis imaging experiments were carried out using the apoTRACE staining kit (Sigma-Aldrich). 24 hours post-PDT, dishes were incubated in the dark with apoTRACE and propidium iodide in the apoTRACE assay medium at concentrations of 75 µg/ml and .5 µg/ml, respectively, for a period of 1 hour. Dishes were then washed with DPBS 1× (CellGrow, Mediatech) and imaged immediately. The apoTRACE dye and propidium iodide were imaged on the hyperspectral confocal microscope. Images were processed using the ImageJ software package. As propidium iodide stains dead cells, and apoTRACE stains cells undergoing apoptosis, only cells displaying both dyes will have undergone apoptotic death at the time of staining. To visualize which cells died via apoptosis, both propidium iodide and apoTRACE fluorescence images were first thresholded using Otsu's method. These images were then compared using a bitwise AND to compute a binary image map showing only apoptotic cells.

### Cryosectioning

The *in vitro* OvCa nodules were sectioned using a HM 550 cryostat (Richard-Allen Scientific). To extract the nodules from the Mattek dishes, the media was entirely removed and immediately replaced by a 4 mm layer of O.C.T. compound (Optical Cutting Temperature, Tissue-Tek, Sakura Finetek). Dishes were placed over a bed of dry ice and the O.C.T. compound was allowed to fully solidify before being transferred to a −80°C freezer. The following day, a portion of the Matted dish plastic side-wall was sliced off using a razor blade (VWR). The thin pointed blade of an X-acto knife was then inserted between the bottom of the O.C.T. disk and the top of the Mattek plastic bottom rim. The blade tip was carefully walked around the circumference of the dish, with great care taken to ensure the blade did not disturb the frozen Matrigel layer. The blade was then gently twisted to separate the O.C.T. disk and its embedded Matrigel layer from the Mattek plate. This block of O.C.T. was then trimmed, the frozen Matrigel block was vertically cut, and pieces of the Matrigel block were laid into cryomolds (15 mm×15 mm×5 mm, Tissue-Tek) partially filled with O.C.T. compound. These cryomolds were immediately placed atop a bed of dry ice, frozen, sealed to lock out moisture, and placed in a −80°C freezer overnight. Prior to cryosectioning, cryomolds were brought to −20 C and allowed to equilibrate for 30 minutes. Frozen samples were sliced into 30 um thick slices, placed on coverglass, mounted with a #1 coverslip to prevent drying, and immediately imaged on the confocal hyperspectral microscope.

### Time-Lapse Optical Coherence Tomography (TL-OCT)

A highly sensitive, label-free cross-sectional imaging technology, TL-OCT can readily visualize entire OvCa nodules, and is thus an ideal method for following the long-term longitudinal three-dimensional structural response to therapy. The TL-OCT system has been described in detail elsewhere [26]. Briefly, the 15 mW broadband output (855 nm centered, 135 nm bandwidth) of a Broadlighter D855HP2 (Superlum) was fiber-coupled into an 80∶20 fiber interferometer (AC Photonics). The 80% arm of the interferometer was sent to a reference arm, while the 20% arm was coupled into a home-built beam scanning inverted microscope (Axiovert 200 M, Zeiss). A 0.15NA Zeiss 5× EC Plan Apo objective lens focused the beam onto the sample. Light reflected from the sample was passed back into the fiber interferometer, and the final interfered light was sent to a home-built spectrometer based on a fast line scan camera (L104k-2048, Basler). The spectral interferogram was read by a fast frame grabber card (1428, National Instruments) and analyzed by custom software developed using Labview and the Intel Performance Primitives Library. Offline image analysis and rendering was accomplished using Matlab. A temperature-controlled microincubation chamber (DH-35i, Warner Instruments) was used for all time-lapse imaging experiments. To maintain normal culture pH, the chamber was pumped just above positive pressure with a 5% CO_2_, 95% air gas mixture (Airgas).

### Chemotherapy and Photodynamic Therapy

To simulate *i.p.* chemotherapy, carboplatin was added to 2% Matrigel supplemented media and incubated for 72 hours at a dose of 1 µM. This dose was calculated from the therapeutic maximum *i.p.* dose of 400 mg/m^2^ based on the culture surface area [18], [27]. To simulate *i.p.* PDT, BPD and EtNBS were added to complete media. Nodules were incubated with 240 nM BPD for 1.5 hours prior to irradiation, following a previously established protocol [18], [27]. The optimal EtNBS concentration was determined through a toxicity screen where nodules grown on a 24 well plate were incubated with a range of EtNBS doses.

PDT was carried out using a fiber-coupled illumination stage. Following incubation, photosensitizer-containing media was removed and replaced with 2% Matrigel-supplemented media with irradiation performed immediately. BPD-PDT was carried out using light from a 690 nm high-powered diode laser (Model 7401, HPD). All EtNBS-PDT experiments, with the sole exception of the hypoxia experiments discussed below, used the output of a 532 nm-pumped tunable dye laser set to 652 nm (600 Series Dye Module, Laserscope). These hypoxia EtNBS-PDT experiments were carried out using a 670 nm diode laser (Model 7401, HPD). To compensate for the small change in EtNBS absorption between 652 and 670 nm, a calculation was carried out using the known laser and EtNBS spectra, resulting in a 5% relative increase in the 670 nm fluence rate. Control experiments found no difference in EtNBS-PDT efficacy or light-induced toxicity between the adjusted 670 nm and original 652 nm irradiances.

To investigate the ability of EtNBS to achieve cytotoxicity under severe hypoxia, a plexiglass chamber was fabricated to feed cultures N_2_ at slight positive pressure. 3.5 hours following EtNBS administration, day 13 plates were placed inside the chamber atop a heating plate and kept at 37°C under 100% N_2_ or CO_2_ for one hour. No cytotoxicity was observed as a result of this incubation. At the end of 4.5 hours, the photosensitizer-containing media was replaced with 2% Matrigel-supplemented complete media which had been bubbled with 100% N_2_ or CO_2_ for five minutes. Plates were treated using the 670 nm diode laser inside the gas chamber. Following treatment, plates were removed from the chamber and returned to an incubator.

## Supporting Information

Figure S1 **Visualizing apoptosis in EtNBS-PDT treated OvCa model nodules.** Cultures were stained with both propidium iodide and apoTRACE. (A) Confocal fluorescence image of propidium iodide-stained OvCa nodules, showing widespread destruction of cancer cells. (B) Binary image map revealing which cells died via apoptosis. Nearly all non-viable cells underwent apoptosis following 10 J/cm^2^ EtNBS-PDT.
(TIFF)

Video S1 **Time-Lapse confocal movie of EtNBS uptake over the course of 4.5 hours.** Day 13 nodules were incubated with EtNBS and visualized using confocal microscopy during the incubation process. EtNBS immediately binds to the surface of the nodules and rapidly diffuses throughout the large nodules over the course of a few hours. Small nodules fill with EtNBS within 3 hours, while the largest nodule in the image takes nearly all 4.5 hours to experience complete EtNBS uptake. Note that scattering of both the excitation light and emitted fluorescence significantly reduces the apparent EtNBS concentration visualized at the center of large OvCa nodules. The movie dimensions are 1.23 mm×1.23 mm.
(MP4)

Video S2 **Time-lapse optical coherence tomography (TL-OCT) of the treatment response of day 13 nodules following EtNBS-PDT.** Each frame of the move is a single xz cross-sectional image through three OvCa nodules. A fraction of the entire depth profile through the Matrigel is shown. The three nodules are seen to sit in the Matrigel bed, and the movie starts immediately following EtNBS-PDT. The nodules are observed to burst open from the inside, rapidly filling with highly scattering apoptotic bodies within five hours following treatment. Cells continue to apoptose over the next several hours, and apoptotic bodies are observed spilling out onto the Matrigel surface. Shaking of the thick Matrigel bed during image acquisition causes the small amount of image warping in the movie. The opening and spilling process seen here explains why nodules treated with EtNBS-PDT at high fluences appear larger than nodules treated at low fluences when viewed with a confocal microscope (Figure 7): by spilling out on the Matrigel bed, the dying nodule spreads out over the gel surface. The movie dimensions are 2.13 mm×0.678 mm.
(MP4)
