# Supplementary figures and images for: Killing Hypoxic Cell Populations in a 3D Tumor Model with EtNBS-PDT
Source: PLoS One. 2011 Aug 18;6(8):e23434. doi: 10.1371/journal.pone.0023434 (PMC3158086; doi:10.1371/journal.pone.0023434)

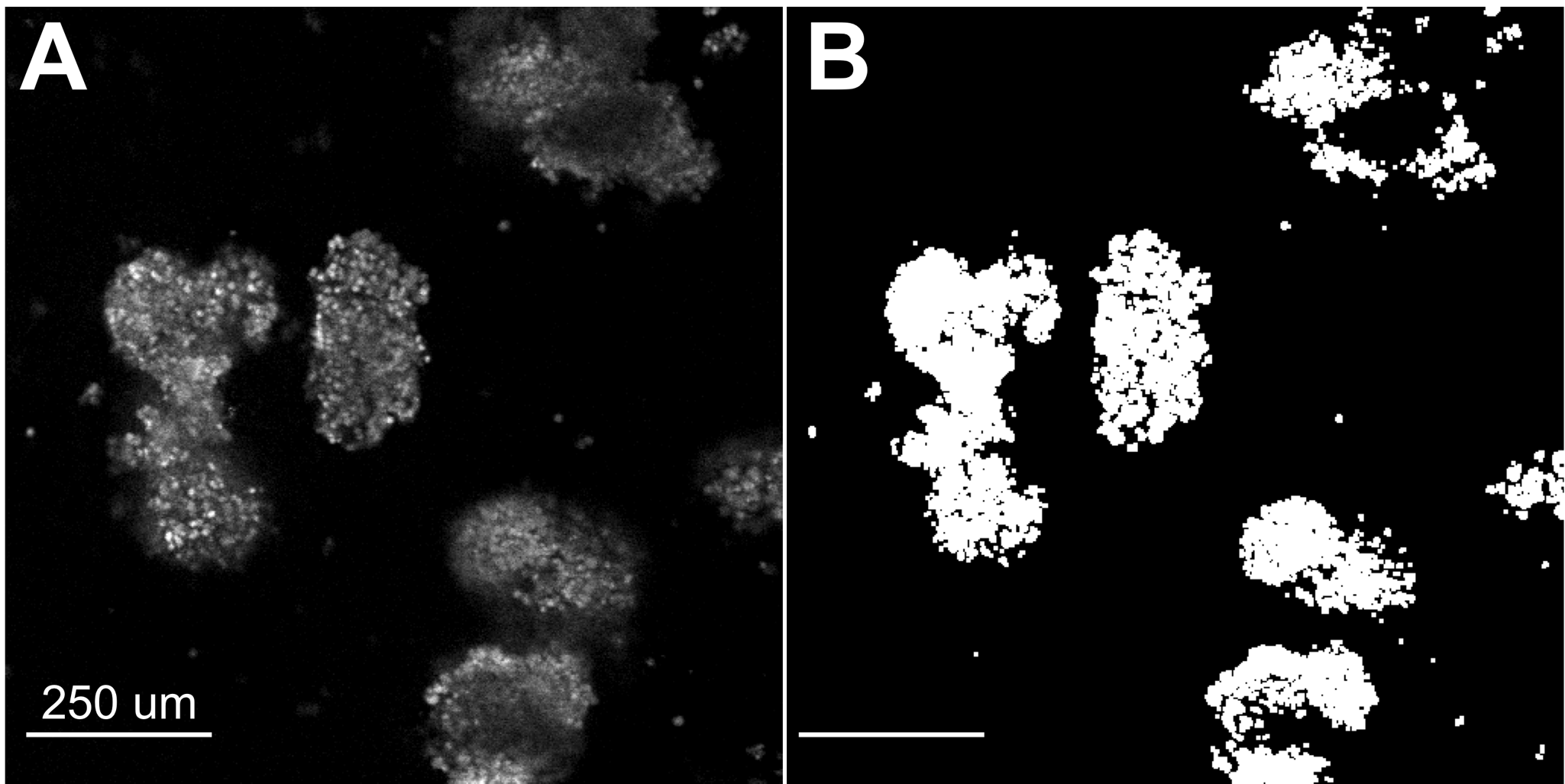

Supplement: Figure S1 — Visualizing apoptosis in EtNBS-PDT treated OvCa model nodules. Cultures were stained with both propidium iodide and apoTRACE. (A) Confocal fluorescence image of propidium iodide-stained OvCa nodules, showing widespread destruction of cancer cells. (B) Binary image map revealing which cells died via apoptosis. Nearly all non-viable cells underwent apoptosis following 10 J/cm2 EtNBS-PDT. (TIFF) [file pone.0023434.s001.tiff]
